# A unified annotation scheme for class D β-lactamases

**DOI:** 10.1128/aac.00562-25

**Published:** 2025-07-21

**Authors:** Malcolm G. P. Page

**Affiliations:** 1 University of Basel 27209, Basel, Switzerland; University of Fribourg, Fribourg, Switzerland

**Keywords:** beta-lactamase

## Abstract

A standardized scheme for numbering and annotation of secondary structure features has been proposed for class D β-lactamases (F. Attana, S. Kim, J. Spencer, B. I. Iorga, et al., Antimicrob Agents Chemother 69:e00150-25, 2025, https://doi.org/10.1128/aac.00150-25). This will significantly facilitate the comparison of biochemical and biophysical studies performed on the disparate members of this class of enzymes and improve communication in the field.

## COMMENTARY

Beta-lactamases represent one of the most important mechanisms of bacterial resistance toward β-lactam antibiotics, such as penicillins, cephalosporins, and carbapenems, rendering them inactive by hydrolyzing the β-lactam moiety of these drugs (1). The resistance afforded by these enzymes can be amplified by additional mechanisms that decrease the lethality of the antibiotics, including mutated or alternative penicillin-binding proteins (2–6) and changes that restrict the net influx of the antibiotic (7). The latter may include mutations of residues in the outer membrane porins, mutations in regulatory systems that result in low expression of these proteins, and loss of the porin genes (8, 9), as well as mutations in the tripartite RND family efflux systems, or their regulatory systems, that result in increased activity of these pumps (10, 11).

The β-lactamases have been classified on a functional basis, according to their specificity for various substrates and sensitivity to inhibitors (12, 13), and on a molecular basis, according to their primary protein sequence (14). Four molecular classes, A, B, C, and D, are generally recognized, and comprehensive standardized numbering schemes have been proposed for the enzymes in classes A (15), B (16, 17), and C (18). Couture et al. (19) proposed a consensus numbering system for class D β-lactamases, but this was based on only five sequences. The class D serine β-lactamases have proliferated extensively since then, with more than 1,400 variants recorded in the beta-lactamase database (20). The article by Attana et al. (21) in this journal provides an updated basis for numbering primary amino acid sequences and a simplified method for the annotation of secondary structure features for the class D enzymes. The four molecular classes differ in the mechanisms by which they hydrolyze their substrates (22): enzymes of classes A, C, and D employ a mechanism in which a nucleophilic serine residue in the active site is transiently acylated by the antibiotic moiety, while the class B enzymes utilize one or two zinc ions in their active site to activate β-lactam substrates and water to drive hydrolysis. Originally identified by their ability to readily hydrolyze oxacillin and analogs (12), the class D enzymes have become of great clinical significance owing to the ability of some variants to inactivate advanced generation cephalosporins and carbapenems (23). The first crystal structure of OXA-10 showed that the overall fold of class D enzymes was similar to those of class A and C β-lactamases (24). Subsequent studies have revealed that the class D enzymes differ from the other serine enzymes in that a lysine in the active site is carbamylated (25, 26, 27). The carbamylated lysine is believed to act as a general acid/base in both acylation and deacylation steps of the hydrolytic mechanism by which class D enzymes catalyze β-lactam hydrolysis.

Attana et al. (21) performed a structural alignment of 21 enzymes representing each of the class D subfamilies listed in the Protein Data Bank. The alignment was used to generate a Hidden Markov Model profile that enables a researcher to align a query sequence with the reference sequence, specifically that of OXA 48, in the numbering scheme. Insertions in the query sequence relative to OXA-48 are identified by lowercase letters (e.g., 216a and 216b), while deletions will result in skipped numbers. The profile also allows for a consensus annotation to label the conserved secondary structure elements in the numerous structures of class D enzymes that are now available, thereby eliminating inconsistencies in structure assignment that have arisen from different determination methods and assignment algorithms. Attana et al. (21) have automated the process to run via a Python-based workflow that can be accessed online using Google Colab (28), and the article provides tutorials to facilitate the use of the program. The work provides a standardized basis for molecular studies that will facilitate comparisons of homologous residues across the plethora of class D enzymes.
